# Coronary computed tomography angiography as a screening tool for moderate-high risk asymptomatic type 2 diabetes mellitus patients

**DOI:** 10.3389/fcvm.2022.974294

**Published:** 2022-08-09

**Authors:** Qiaolu Liu, Jianfeng Qiu, Shuxin Sun, Xiaoqiang Wang, Zhanguo Sun, Huihui Zhao

**Affiliations:** ^1^Department of Radiology, Affiliated Hospital of Jining Medical University, Jining, China; ^2^Department of Radiology, Shandong First Medical University and Shandong Academy of Medical Sciences, Tai’an, China; ^3^The Second Affiliated Hospital of Shandong First Medical University, Tai’an, China; ^4^Shandong Engineering Laboratory, Medical Imaging and Quantitative Analysis, Tai’an, China

**Keywords:** coronary computed tomography angiography (CCTA), type 2 diabetes mellitus, coronary heart disease, risk stratification prediction, United Kingdom prospective diabetes study

## Abstract

**Background:**

There are few data on the clinical significance of coronary computed tomography angiography (CCTA) in asymptomatic type 2 diabetes mellitus (T2DM) patients. We performed a retrospective study to evaluate coronary heart disease (CHD) screening in asymptomatic patients with T2DM using CCTA and CHD risk stratification prediction.

**Materials and methods:**

Data from 141 T2DM patients (58 ± 8 years, 57% males) without known symptoms suggestive of CHD who underwent CCTA were retrospectively analyzed. The patients were classified into three subgroups based on United Kingdom prospective diabetes study (UKPDS) CHD risk stratification prediction. Seventy-four patients without diabetes mellitus and CHD who underwent CCTA successively were chosen as the control group. The segment involvement score (SIS), segment stenosis score (SSS), stenosis coefficient (SC), severe proximal plaque (SPP) positive ratio and CCTA-adapted Leaman score (CT-LeSc) based on CCTA data were evaluated and compared among the groups.

**Results:**

Compared with the patients in the control group, patients in the moderate-high risk DM groups had higher scores on the SIS, SSS, SC, CT-LeSc, and a higher SPP positive ratio (all *p*-values < 0.001), and no difference was observed between the low-risk group and the control group (*p* = 0.136, *p* = 0.088, *p* = 0.0.067, *p* = 0.225, *p* = 1.000, respectively). Compared with patients in the control group, the patients in the moderate-high risk DM groups had increased odds of SIS > 3 [odds ratio (OR) = 6.557, *p* < 0.001; OR = 4.455, *p* < 0.001, respectively], SSS > 5 (OR = 5.727, *p* < 0.001; OR = 5.144, *p* < 0.001, respectively), CT-LeSc > 8.7 (OR = 3.780, *p* = 0.001; OR = 2.804, *p* = 0.007, respectively), and obstructive stenosis (OR = 7.233, *p* < 0.001; OR = 5.787, *p* < 0.001, respectively).

**Conclusion:**

The moderate-high CHD risk patients had increased odds of obstructive coronary artery stenosis, and the distribution of coronary artery stenosis was more extensive and more severe in that group compared to the patients without diabetes mellitus and CHD. CHD can be effectively screened in moderate-high risk asymptomatic T2DM patients using CCTA.

## Introduction

Diabetes will be a significant problem in the future, from 537 million patients affected worldwide in 2021 to 783 million people projected by 2045, representing an approximately 46% growth (1). Many studies in the literature have shown a clear correlation between type 2 diabetes mellitus (T2DM) and the risk of coronary heart disease (CHD) (2–5). Comparing with non-diabetics patients, patients with T2DM suffer from CHD at a much younger age (6). Additionally, T2DM patients are at a higher risk for developing CHD, about four times that of their peers without diabetes, even if they lack any pertinent symptoms (7, 8).

Currently, coronary computed tomography angiography (CCTA) is widely used to evaluate the degree of coronary artery stenosis and the characteristics of atherosclerotic plaque (9–12). Although invasive coronary angiography (ICA) is still the gold standard for the diagnosis of coronary artery disease, CCTA is increasingly becoming a viable non-invasive alternative. In addition to providing a faster and possibly more cost-effective way to assess the patients at a moderate CHD fatal risk, CCTA also avoids the risks associated with invasive surgery. As a more advanced technology, CCTA now has enough temporal and spatial resolution to evaluate the coronary artery tree and even the distal lumen and has allowed for the accurate assessment of the stenosis severity and atherosclerotic plaque composition (13). Compared with ICA, CCTA has good sensitivity, specificity and negative predictive value in the detection of CHD, but the positive predictive value of CCTA is lower than that of ICA (14). There are few data on the clinical significance of CCTA in asymptomatic T2DM patients (15, 16).

This study was performed to investigate a method of CHD screening in asymptomatic T2DM patients using CCTA and CHD fatal risk stratification prediction. We compared the CCTA scores among different risk groups and the control group to clarify the role of CCTA in asymptomatic T2DM patient screening.

## Materials and methods

### Patients

A total of 149 continuously T2DM patients at the Affiliated Hospital of Jining Medical University without known symptoms suggestive of CHD who underwent CCTA from July 2019 to December 2019 were enrolled in the study. All T2DM patients had at least one other cardiovascular risk factor.

Type 2 diabetes mellitus was confirmed according to the criteria of the American Diabetes Association (17): Glycated hemoglobin (HbA1c) levels ≥6.5%, fasting blood glucose levels ≥7.0 mmol/l and/or a post-challenge blood glucose level ≥11.1 mmol/l (2 h after a 75 g oral glucose load) or the current use of hypoglycemic treatment. The symptom asymptomatic status of the patients was evaluated using the Rose questionnaire for angina. Patients without CHD were defined as asymptomatic.

The cardiovascular risk factors included the following: hypertension (defined as blood pressure ≥140/90 mmHg or the use of antihypertensive medication) (18), dyslipidemia (defined as a total cholesterol level ≥5.2 mmol/l or treatment with lipid-lowering drugs) (19), smoking, obesity (body mass index ≥28 kg/m^2^) (20) or lack of exercise (defined as not exercise regularly at least three times a week), and family history of myocardial infarction in first-degree relatives.

The exclusion criteria were as follows: type 1 diabetes; known or suspected CHD; abnormal resting electrocardiographic results; history of prior myocardial infarction, history of coronary artery bypass grafting or stenting; and incomplete clinical data.

One hundred and forty-eight other continuous non-T2DM non-CHD patients at the Affiliated Hospital of Jining Medical University, who were found to have slight electrocardiogram abnormalities and underwent CCTA to exclude any coronary artery disease, from December 2019 to January 2020 were chosen as the control group.

**FIGURE 1:**
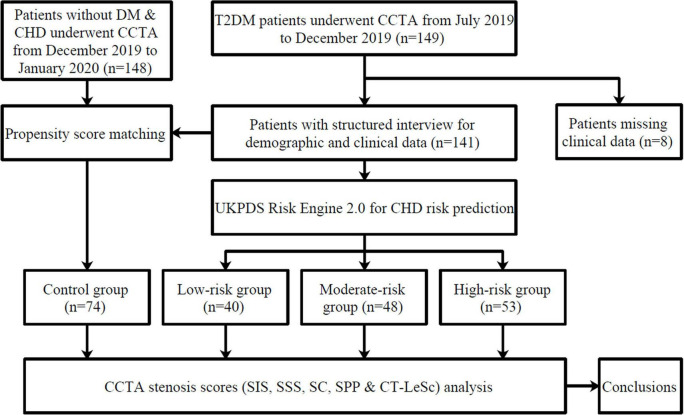
A flow diagram of the study.

A structured interview was performed to record the demographic and clinical data. Seventy-four patients were finally included in the control group after propensity score matching due to the difference in age, triglycerides, and high-density lipoprotein (HDL) cholesterol between the control group and the DM group. The flow diagram of the study is shown in Figure 1.

### Coronary heart disease fatal risk stratification prediction

The CHD fatal risk stratification of T2DM patients was forecasted by the United Kingdom prospective diabetes study (UKPDS) risk engine 2.0.^1^ The UKPDS is a group of clinical trials, epidemiological analyses and health-modeling studies with an influence that can be assessed across a broad range of health domains. The UKPDS made the risk of age, hyperglycemia, elevated blood pressure, adverse blood lipids and smoking contributions more clear. Equations were developed, combined and incorporated into the UKPDS risk engine. The UKPDS risk engine provides risk estimates in individuals with T2DM not known to have heart disease (21, 22).

The following characteristics were used to calculate the patients’ CHD fatal risk: age, duration of diabetes, sex, ethnicity, atrial fibrillation, smoking history, HbA1c, systolic blood pressure, total cholesterol, and HDL cholesterol. A positive smoking history was defined as current smoking or cessation of smoking within 3 months.

### Multidetector CT scan protocol and image reconstruction

Coronary computed tomography angiography was performed using a dual source CT scanner (SOMATOM Definition Flash dual-source; Siemens Medical Solutions, Erlangen, Germany) following standard guidelines (23).

During the CCTA acquisition, 50–80 ml iodinated contrast (Ultravist 370, Bayer Schering, Berlin, Germany) was injected based on the individual’s weight, followed by a 30 ml saline flush, the injection rate was 5–7 ml/s. A retrospective ECG-gated spiral scan was performed covering the region immediately beneath the aortic arch to the apex of the left ventricle during an inspiratory breath hold of 10–20 s. The scan parameters were as follows: gantry rotation for 330–420 ms, spiral imaging with retrospective ECG gating and automatic dose modulation, 750–850 mA, 100 or 120 kV and 0.75 mm slice thickness, 128 × 0.6 mm collimation. A multisegment algorithm was used to reconstruct overlapping images, typically at 75% of the cardiac cycle in central diastole. If motion artifacts were present, additional reconstructions were made at different points of the R-R interval, as needed. All reconstructed datasets were sent to a dedicated workstation (syngo.*via* VA 20B, Siemens Healthcare, Erlangen, Germany) for post-processing and three dimensional reconstruction.

### Coronary stenosis analysis

The coronary arteries were divided into 18 segments following the Society of Cardiovascular Computed Tomography guidelines (24). Each segment was examined for coronary plaques. The structures of >1 mm^2^ and adjacent to or within the coronary artery lumen that could be clearly separated from the vessel lumen were scored as a coronary plaque (25). Each coronary segment was scored individually for the presence of plaque, and any stenosis was visually quantified. Coronary stenosis was assessed by the following clinical coronary plaque scores: the segment stenosis score (SSS); segment involvement score (SIS); stenosis coefficient (SC); and severe proximal plaque (SPP) (26).

**FIGURE 2:**
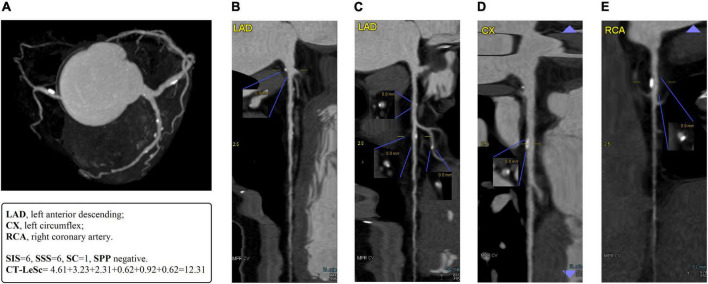
A case example of 55-years-old man with T2DM for 10 years. **(A)** The MIP of coronary tree with blood pool removal shows right dominance and six non-obstructive stenosis segments in LAD, D1, LCx, and RCA. **(B)** The straightened MPR of LAD shows a mixed plaque with mild lumen stenosis in LM. The CT-LeSc = 5 × 1.5 × 0.615 (weighting for localization × type of plaque × stenosis severity) = 4.61. **(C)** The straightened MPR of LAD shows mixed plaques with mild lumen stenosis in proximal and mid LAD, a calcified plaque with mild lumen stenosis in D1. The CT-LeSc = 3.23 (3.5 × 1.5 × 0.615), 2.31 (2.5 × 1.5 × 0.615), 0.62 (1 × 1 × 0.615), respectively. **(D)** The straightened MPR of LCx show a calcified plaque with mild lumen stenosis in proximal LCx. The CT-LeSc = 0.92 (1.5 × 1 × 0.615). **(E)** The straightened MPR of RCA show a calcified plaque with mild lumen stenosis in proximal RCA. The CT-LeSc = 0.62 (1 × 1 × 0.615). The total CT-LeSc of this patient = 4.61 + 3.23 + 2.31 + 0.62 + 0.92 + 0.62 = 12.31. There are six segment coronary plaques, so the SIS of this patient is 6. Mild lumen stenosis is found in all 6 plaques with score 1 of SSS, the SSS of this patient is 6. The SC = SSS÷SIS = 1. There is no severe proximal plaque in the proximal of LAD, LCx, and RCA, so the SPP is negative. MIP, maximum density projection; MPR, multiplanar reconstruction; LAD, left anterior descending; LM, left main; D1, 1st diagonal; LCx, left circumflex; RCA, right coronary artery; CT-LeSc, CCTA-adapted Leaman score; SIS, segment involvement score; SSS, segment stenosis score; SC, stenosis coefficient; SPP, severe proximal plaque.

The SIS reflected the plaque distribution and was calculated as the total number of coronary artery segments exhibiting plaque, irrespective of the degree of luminal stenosis within each segment (scores from 0 to 18). The SSS was used as a measure of the overall coronary artery plaque burden. Each individual coronary segment was graded as having no to severe plaque (scores from 0 to 3) based on the extent of the obstruction of the coronary luminal diameter. Then, the extent scores of all 18 individual segments were summed to yield a total score. The SC was defined as the ratio of the SSS to SIS (the SC was defined as 0 when the SIS was 0), which reflected the degree of artery stenosis. The presence of any severe plaque in the left main or proximal portion of the left anterior descending, left circumflex and right coronary arteries was defined as SPP positive. A vessel stenosis greater than 70% was defined as a severe plaque. An obstructive stenosis (OS) lesion was defined as a ≥50% reduction in the diameter of the lumen (26, 27). A case example is shown in Figure 2.

### Coronary computed tomography angiography-adapted Leaman score

Coronary computed tomography angiography-adapted Leaman score (CT-LeSc) was used to quantify total coronary atherosclerotic burden with information regarding localization, type of plaque and degree of stenosis. The methodology for the CT-LeSc is presented in Table 1. Three sets of weighting factors were used: (1) localization of the coronary plaques, accounting for dominance, (2) type of plaque, with a multiplication factor of 1 for calcified plaques and of 1.5 for non-calcified and mixed plaques, (3) degree of stenosis, with a multiplication factor of 0.615 for non-OS and a multiplication factor of 1 for OS lesions. The CT-LeSc on a patient level was calculated as the sum of the partial CT-LeSc of all evaluable coronary segments (28). A case example is shown in Figure 2.

**TABLE 1:** Coronary computed tomography angiography-adapted Leaman score (CT-LeSc) weighting factors.

	Right dominance	Left dominance	Balanced
**Coronary segments**			
RCA proximal	1	0	0.5
RCA mid	1	0	0.5
RCA distal	1	0	0.5
PDA	1	na	0.5
Left main	5	6	5.5
LAD proximal	3.5	3.5	3.5
LAD mid	2.5	2.5	2.5
LAD distal	1	1	1
1st diagonal	1	1	1
2nd diagonal	0.5	0.5	0.5
LCx proximal	1.5	2.5	2
1st obtuse marginal	1	1	1
LCx distal	0.5	1.5	1
2nd obtuse marginal	1	1	1
PDA from LCA	na	1	na
PL branch from LCA	na	0.5	0.5
PL branch from RCA	0.5	na	na
Intermediate branch	1	1	1
**Stenosis severity**			
Obstructive stenosis	1		
Non-obstructive stenosis	0.615		
**Plaque composition**			
Non-calcified or mixed	1.5		
Calcified	1		

RCA, right coronary artery; PDA, posterior descending artery; LAD, left anterior descending; LCx, left circumflex; PL, postero-lateral.

The examination results were independently interpreted and summarized by two doctors with more than 5 years of CCTA diagnosis experience. If there was any difference, the final results were decided by the two doctors after consultation and discussion.

### Statistical analysis

Continuous variables with a normal distribution are presented as the mean ± standard deviation; non-normal variables are presented as the median (interquartile range). Categorical variables were expressed as frequencies. Continuous variables were compared by the Mann–Whitney *U* test or Kruskal–Wallis test. Differences in the categorical variables were assessed using the χ^2^ test. The association of T2DM and CCTA findings was analyzed by binary logistic regression. All statistical analyses were performed using SPSS 26.0 software (SPSS, Inc., Chicago, IL, United States), and *p* < 0.05 was considered statistically significant.

## Results

Of the 149 T2DM patients who underwent CCTA, 8 (no HbA1c or HDL cholesterol results) were excluded and 141 patients (80 males, 57%) were evaluated in the current study. Seventy-four patients were finally included in the control group after propensity score matching due to the difference in age, triglycerides and HDL cholesterol between the control group and the DM group.

In the T2DM patients the median course of disease was (4, 15) 9 years, and 42 (30%) were current smokers. All patients were of Asian ethnicity and had no atrial fibrillation history. Seventy-four non-DM and non-CHD patients (38 males, 51%) were included in the control group, and 17 (23%) were current smokers. There were no significant between-group differences with respect to sex or current smoking ratio (*p* = 0.451, *p* = 0.287, respectively). Further patient demographics and characteristics are presented in Table 2.

**TABLE 2:** Clinical and biochemical characteristics of the study population.

Characteristics	Control group	DM group	*P*-value (*p* < 0.05)
*N*	74	141	–
^†^Male gender	38 (51%)	80 (57%)	χ^2^ = 0.569 *p* = 0.451^a^
^‡^Age (years)	65 ± 13	58 ± 8	*U* = 4959.500 *p* = 0.552^b^
^†^Current smoker	17 (23%)	42 (30%)	χ^2^ = 0.132 *p* = 0.287^a^
^§^ Duration of diabetes (years)	–	9 (4, 15)	–
^‡^SBP (mmHg)	139 ± 19	136 ± 18	U = 4864.500 p = 0.416^b^
^§^ Triglycerides (mmol/l)	1.3 (0.9, 1.7)	1.5 (1.0, 2.1)	*U* = 6062.500 *p* = 0.051^b^
^‡^Total cholesterol (mmol/l)	4.5 ± 1.0	4.5 ± 1.1	*U* = 5175.000 *p* = 0.923^b^
^‡^HDL cholesterol (mmol/l)	1.2 ± 0.2	1.2 ± 0.3	*U* = 5093.000 *p* = 0.775^b^
^§^ FBG (mmol/l)	5.4 (4.8, 5.8)	7.1 (5.7, 8.9)	*U* = 8448.500 *p* < 0.001^b^*
^§^ HbA1c (%)	–	8.7 (7.4, 10.3)	–

^†^Data are expressed as *n* (%). ^‡^Data are expressed as mean ± standard deviation. ^§^Data are expressed as the median (interquartile range). *^a^p*-value by χ^2^ test. ^b^*p*-value by Mann–Whitney *U* test. *p < 0.05. DM, diabetes mellitus; SBP, systolic blood pressure; HDL, high-density lipoprotein; FBG, fasting blood glucose.

According to the CHD fatal risk in 10 years predicted by the UKPDS risk engine, the DM group was divided into three subgroups: the low-risk group (fatal risk <7.5%), moderate-risk group (fatal risk 7.5–15%), and high-risk group (fatal risk >15%).

No significant differences were observed with respect to male sex or current smoking ratio in multiple groups (*p* = 0.184, *p* = 0.202, respectively). Further patient demographics and characteristics are presented in Table 3.

**TABLE 3:** Comparisons of the clinical and biochemical characteristics among the DM subgroups.

Characteristics	Low-risk group	Moderate-risk group	High-risk group	*P*-value (*p* < 0.05)
*N*	40	48	53	–
^†^Male gender	19 (48%)	26 (54%)	35 (66%)	χ^2^ = 3.387 *p* = 0.184^a^
^‡^Age (years)	51 ± 5	58 ± 6	63 ± 8	*H* = 50.548 *p* < 0.001^b^*
^†^Current smoker	8 (20%)	18 (38%)	16 (30%)	χ^2^ = 3.201 *p* = 0.202^a^
^§^ Duration of diabetes (years)	1 (6, 9)	3 (10, 13)	15 (7, 20)	*H* = 20.548 *p* < 0.001^b^*
^‡^SBP (mmHg)	128 ± 16	134 ± 17	143 ± 17	*H* = 15.734 *p* < 0.001^b^*
^§^ Triglycerides (mmol/l)	1.2 (0.9, 1.7)	1.5 (0.9, 2.1)	1.6 (1.2, 2.2)	*H* = 4.552 *p* = 0.103^b^
^‡^Total cholesterol (mmol/l)	4.0 ± 1.0	4.7 ± 1.1	4.7 ± 1.1	*H* = 11.145 *p* = 0.004^b^*
^‡^HDL cholesterol (mmol/l)	1.1 ± 0.2	1.2 ± 0.3	1.1 ± 0.2	*H* = 3.009 *p* = 0.222^b^
^§^ FBG (mmol/l)	6.6 (5.3, 8.3)	7.3 (6.4, 8.6)	7.4 (6.1, 9.7)	*H* = 6.985 *p* = 0.030^b^*
^‡^HbA1c (%)	8.1 ± 1.8	9.1 ± 2.2	9.6 ± 2.0	*H* = 13.749 *p* = 0.001^b^*

^†^Data are expressed as *n* (%). ^‡^Data are expressed as the mean ± standard deviation. ^§^Data are expressed as the median (interquartile range). ^*a*^*p*-value by χ^2^ test. ^b^*p*-value by Kruskal–Wallis test. *p < 0.05. SBP, systolic blood pressure; HDL, high-density lipoprotein; FBG, fasting blood glucose.

### The cardiovascular risk factors of type 2 diabetes mellitus patients

There were one hundred (71%) patients with hypertension, 35 (25%) patients with dyslipidemia, 42 (30%) current smokers, 40 (28%) patients with obesity or lack of exercise, and 30 (21%) patients with family history of myocardial infarction in first-degree relatives. Seventy-seven (55%) patients had more than one cardiovascular risk factor (Table 4).

**TABLE 4:** The cardiovascular risk factors of T2DM patients.

Cardiovascular risk factors	DM patients (*n* = 141)
Hypertension	100 (71%)
Dyslipidemia	35 (25%)
Current smoker	42 (30%)
Obesity or lack of exercise	40 (28%)
Family history of myocardial infarction	30 (21%)
Patients with more than one risk factors	77 (55%)

Data are expressed as *n* (%).

### Coronary stenosis analysis

A total of 788 coronary stenosis segments were found in 215 patients, including 629 segments in 141 T2DM patients and 159 segments in 74 patients of the control group. Among 215 patients, 155 (72%) had stenosis in the proximal segment of the left anterior descending, accounting for 20% of all stenosis segments (Table 5).

**TABLE 5:** Coronary stenosis segments in different groups.

	Control group	Low-risk group	Moderate-risk group	High-risk group
Coronary stenosis segments	*n* = 159	*n* = 132	*n* = 235	*n* = 262
RCA proximal	18 (11%)	22 (17%)	32 (14%)	34 (13%)
RCA mid	17 (11%)	14 (11%)	24 (10%)	26 (10%)
RCA distal	13 (8%)	8 (6%)	17 (7%)	14 (5%)
PDA	3 (2%)	3 (2%)	9 (4%)	7 (3%)
Left main	12 (8%)	8 (6%)	18 (8%)	24 (9%)
LAD proximal	37 (23%)	32 (24%)	38 (16%)	48 (48%)
LAD mid	25 (16%)	15 (11%)	22 (9%)	27 (10%)
LAD distal	1 (1%)	0	2 (1%)	2 (1%)
1st diagonal	7 (4%)	8 (6%)	18 (8%)	21 (8%)
2nd diagonal	1 (1%)	0	6 (3%)	3 (1%)
LCx proximal	16 (10%)	15 (11%)	27 (11%)	26 (10%)
1st obtuse marginal	5 (3%)	3 (2%)	9 (4%)	13 (5%)
LCx distal	3 (2%)	2 (2%)	5 (2%)	6 (2%)
2nd obtuse marginal	0	0	2 (1%)	2 (1%)
PDA from LCA	0	0	0	0
PL branch from LCA	0	2 (2%)	5 (2%)	5 (2%)
PL branch from RCA	1 (1%)	0	1 (1%)	4 (2%)
Intermediate branch	0	0	0	0
**Stenosis severity**				
Obstructive stenosis	21 (13%)	29 (22%)	107 (46%)	124 (47%)
Non-obstructive stenosis	138 (87%)	103 (78%)	128 (54%)	138 (53%)
**Plaque composition**				
Non-calcified or mixed	139 (87%)	128 (97%)	214 (91%)	234 (89%)
Calcified	20 (13%)	4 (3%)	21 (9%)	28 (11%)

RCA, right coronary artery; PDA, posterior descending artery; LAD, left anterior descending; LCx, left circumflex; PL, postero-lateral.

**FIGURE 3:**
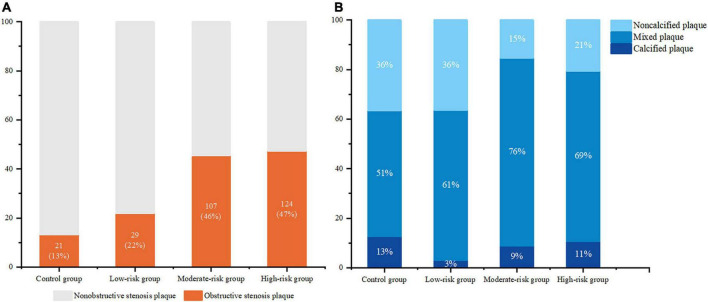
The proportion of obstructive stenosis plaque **(A)** and plaque composition proportion **(B)** in the control group and DM subgroups.

Two hundred and sixty (41%) OS plaques were found in T2DM patients and 21 (13%) OS plaques in control group (Figure 3A).

There were 53 (8%) calcified plaques and 138 (22%) non-calcified plaques in T2DM patients, the others were mixed plaques. The quantity were 20 (13%) and 58 (36%) respectively in the control group (Figure 3B).

The patients in the DM group had significantly higher CCTA stenosis scores (SPP, SIS, SSS, and SC) and CT-LeSc than those in the control group (all *p*-values < 0.001); see Table 6 for details.

**TABLE 6:** Coronary stenosis scores of the study population.

Coronary stenosis scores	Control group	DM group	*P*-value (*p* < 0.05)
*N*	74	141	–
^†^SPP positive	1 (1.4%)	20 (14.2%)	χ^2^ = 14.424 *p* < 0.001^a^*
^‡^SIS	1.0 (0.0, 4.0)	4.0 (2.0, 7.0)	*U* = 7587.000 *p* < 0.001^b^*
^‡^SSS	1.0 (0.0, 4.3)	5.0 (2.0, 9.0)	*U* = 7753.500 *p* < 0.001^b^*
^‡^SC	1.0 (0.0, 1.0)	1.0 (1.0, 1.5)	*U* = 7680.500 *p* < 0.001^b^*
^‡^CT-LeSc	3.23 (0.0, 8.76)	7.56 (3.92, 13.3)	*U* = 7487.500 *p* < 0.001^b^*

^†^Data are expressed as *n* (%). ^‡^Data are expressed as median (interquartile range). *^a^p*-value by χ^2^ test. ^b^*p*-value by Mann–Whitney *U* test. *p < 0.05. DM, diabetes mellitus; SPP, severe proximal plaque; SIS, segment involvement score; SSS, segment stenosis score; SC, stenosis coefficient; CT-LeSc, CCTA-adapted Leaman score.

**FIGURE 4:**
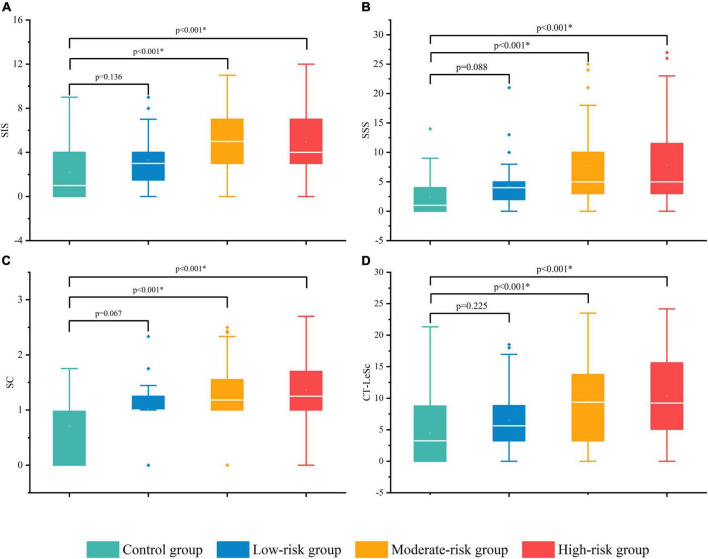
The difference in the CCTA stenosis scores among the control group and DM subgroups. SIS **(A)**, SSS **(B)**, SC **(C)**, and CT-LeSc **(D)**. SIS, segment involvement score; SSS, segment stenosis score; SC, stenosis coefficient; CT-LeSc, CCTA-adapted Leaman score. *p-value < 0.05.

The SIS in the moderate-risk group [5.0 (3.0, 7.0)] and high-risk group [4.0 (2.5, 7.0)] were significantly higher than those in the control group [1.0 (0.0, 4.0)] (*p* < 0.001, *p* < 0.001, respectively), and no difference was observed between the low-risk group [3.0 (1.3, 4.0)] and the control group (*p* = 0.136). No difference was observed among the DM subgroups (see Tables 7, 8 for details). This suggests a wider distribution of coronary artery stenosis in asymptomatic T2DM patients of moderate-high risk than in non-DM non-CHD patients. The difference in the SIS among the groups is shown in Figure 4A.

**TABLE 7:** Comparisons of the coronary stenosis scores among the CHD risk subgroups and the control group.

Coronary stenosis scores	control group	DM group			*P*-value
		Low-risk group	moderate-risk group	high-risk group	(*p* < 0.05)
*N*	74	40	48	53	–
^†^SPP positive	1 (1%)	1 (3%)	8 (17%)	11 (21%)	χ^2^ = 18.196 *p* < 0.001^a^*
^‡^SIS	1.0 (0.0, 4.0)	3.0 (1.3, 4.0)	5.0 (3.0, 7.0)	4.0 (2.5, 7.0)	*H* = 36.953 *p* < 0.001^b^*
^‡^SSS	1.0 (0.0, 4.3)	3.5 (1.3, 5.0)	5.5 (3.0, 10.0)	5.0 (3.0, 12.5)	*H* = 42.155 *p* < 0.001^b^*
^‡^SC	1.0 (0.0, 1.0)	1.0 (1.0, 1.3)	1.2 (1.0, 1.6)	1.3 (1.0, 1.7)	*H* = 43.066 *p* < 0.001^b^*
^‡^CT-LeSc	3.23 (0.00, 8.76)	5.61 (3.23, 9.02)	9.36 (3.23, 13.82)	9.23 (4.90, 16.08)	*H* = 34.613 *p* < 0.001^b^*

^†^Data are expressed as *n* (%). ^‡^Data are expressed as the median (interquartile range). *^a^p*-value by χ^2^ test. ^b^*p*-value by Kruskal–Wallis test. *p < 0.05. SPP, severe proximal plaque; SIS, segment involvement score; SSS, segment stenosis score; SC, stenosis coefficient; CT-LeSc, CCTA-adapted Leaman score.

**TABLE 8:** *Post hoc* multiple comparisons among the CHD risk subgroups and the control group.

Coronary stenosis scores	*P*-value (*p* < 0.05)					
	Group 1–2	Group 1–3	Group 1–4	Group 2–3	Group 2–4	Group 3–4
SPP^a^	1.000	0.005*	0.001*	0.067	0.022*	0.787
SIS^b^	0.136	<0.001*	<0.001*	0.131	0.151	1.000
SSS^b^	0.088	<0.001*	<0.001*	0.109	0.083	1.000
SC^b^	0.067	<0.001*	<0.001*	0.186	0.061	1.000
CT-LeSc^b^	0.225	<0.001*	<0.001*	0.247	0.071	1.000

^a^*p*-value by χ^2^ test. ^b^*p*-value by Kruskal–Wallis test with Bonferroni correction for the *post-hoc* test. *p-value < 0.05. Group 1, control group; Group 2, low CHD risk group; Group 3, moderate CHD risk group; Group 4, high CHD risk group; SPP, severe proximal plaque; SIS, segment involvement score; SSS, segment stenosis score; SC, stenosis coefficient; CT-LeSc, CCTA-adapted Leaman score.

The SSS in the moderate-risk group [5.5 (3.0, 10.0)] and the high-risk group [5.0 (3.0, 12.5)] were significantly higher than that in the control group [1.0 (0.0, 4.3)] (*p* < 0.001, *p* < 0.001, respectively), and no difference was observed between the low-risk group [3.5 (1.3, 5.0)] and the control group (*p* = 0.088). No difference was observed among DM subgroups (see Tables 7, 8 for details). The overall burden of coronary plaque was more severe in the moderate-high risk asymptomatic T2DM patients than in the non-DM non-CHD patients. The difference in the SSS among the groups is shown in Figure 4B.

The SC in the moderate-risk group [1.2 (1.0, 1.6)] and high-risk group [1.3 (1.0, 1.7)] was significantly higher than that in the control group [1.0 (0.0, 1.0)] (*p* < 0.001, *p* < 0.001, respectively), and no difference was observed between the low-risk group [1.0 (1.0, 1.3)] and the control group (*p* = 0.067). No difference was observed among the DM subgroups (see Tables 7, 8 for details). This suggests more severe coronary artery stenosis in the moderate-high risk asymptomatic T2DM patients than in the non-DM and non-CHD patients. The difference in the SC among the groups is shown in Figure 4C.

**FIGURE 5:**
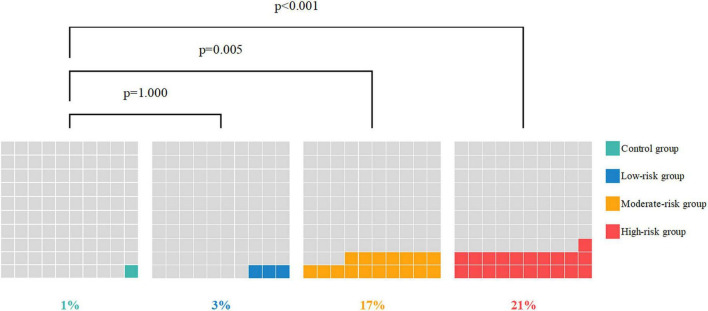
The SPP-positive ratio of the control group and DM subgroups.

The positive SPP ratios in the moderate-risk group (17%) and high-risk group (21%) were significantly higher than that in the control group (1%) (*p* < 0.001, *p* < 0.001, respectively), and no difference was observed between the low-risk group (3%) and the control group (*p* = 1.000). No difference was observed between the moderate and high-risk groups (see Tables 7, 8 for details). Severe proximal coronary artery stenosis was observed in the moderate-high risk asymptomatic T2DM patients compared with the non-DM non-CHD patients, which suggests the poor prognosis of CHD. The difference in the SC among the groups is shown in Figure 5.

The CT-LeSc in the moderate-risk group [9.36 (3.23, 13.82)] and high-risk group [9.23 (4.90, 16.08)] was significantly higher than that in the control group [3.23 (0.00, 8.76)] (*p* < 0.001, *p* < 0.001, respectively), and no difference was observed between the low-risk group [5.61 (3.23, 9.02)] and the control group (*p* = 0.225). No difference was observed among the DM subgroups (see Tables 7, 8 for details). The total coronary atherosclerotic burden in the moderate-high risk asymptomatic T2DM patients was higher than that in the non-DM and non-CHD patients. The difference in the CT-LeSc among the groups is shown in Figure 4D.

There was no difference in any of the four CCTA stenosis scores (SPP, SIS, SSS, and SC) and CT-LeSc between the low-risk group and the control group (*p* = 1.000, *p* = 0.136, *p* = 0.088, *p* = 0.067, and *p* = 0.225, respectively).

**FIGURE 6:**
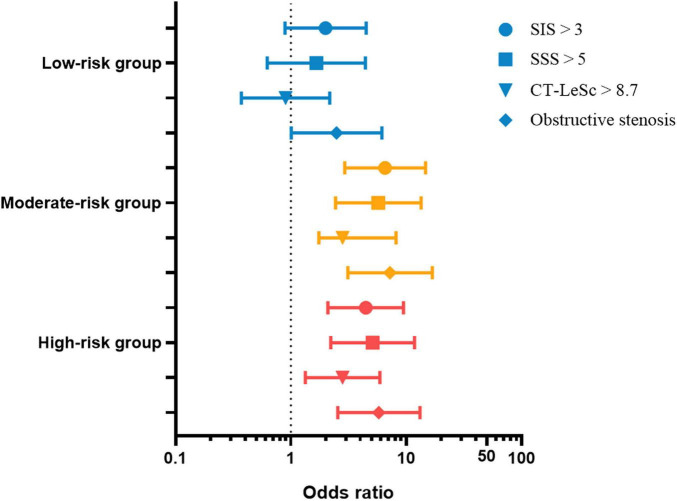
The SIS > 3, SSS > 5, CT-LeSc > 8.7, and obstructive stenosis odds ratio (95% CI) of the DM group and subgroups. SIS, segment involvement score; SSS, segment stenosis score; SC, stenosis coefficient; CT-LeSc, CCTA-adapted Leaman score. The control group was used as the reference group.

Compared with the patients in the control group, the patients in the DM group had an increased risk of SIS > 3 [odds ratio (OR) = 3.979 (2.154–7.348), *p* < 0.001], SSS > 5 [OR = 4.002 (1.942–8.247), *p* < 0.001], CT-LeSc > 8.7 [OR = 2.309 (1.254–4.253), *p* = 0.007], and OS [4.951 (2.457–9.979), *p* < 0.001]. The risk was even higher in the moderate-risk group [OR = 6.557 (2.927–14.690), *p* < 0.001; OR = 5.727 (2.437–13.461), *p* < 0.001; OR = 3.780 (1.751–8.162), *p* = 0.001; OR = 7.233 (3.112–16.812), *p* < 0.001, respectively] and the high-risk group [OR = 4.455 (2.092–9.489), *p* < 0.001; OR = 5.114 (2.214–11.813), *p* < 0.001; OR = 2.804 (1.333–5.899), *p* = 0.007; OR = 5.787 (2.548–13.143), *p* < 0.001, respectively]. The risk in the low-risk group was lower than that in the whole DM group and had no difference with the patients in the control group except OS; see Table 9 and Figure 6 for details.

**TABLE 9:** Binary logistic regression analysis of the CCTA findings.

CCTA findings	Control group	DM group		Low-risk group		Moderate-risk group		High-risk group	
		OR (95% CI)	*P*-value	OR (95% CI)	*P*-value	OR (95% CI)	*P*-value	OR (95% CI)	*P*-value
	Reference								
SIS > 3		3.979 (2.154–7.348)	<0.001*	1.996 (0.888–4.486)	0.095	6.557 (2.927–14.690)	<0.001*	4.455 (2.092–9.489)	<0.001*
SSS > 5		4.002 (1.942–8.247)	<0.001*	1.663 (0.624–4.432)	0.309	5.727 (2.437–13.461)	<0.001*	5.114 (2.214–11.813)	<0.001*
CT-LeSc > 8.7		2.309 (1.254–4.253)	0.007*	0.900 (0.373–2.171)	0.815	3.780 (1.751–8.162)	0.001*	2.804 (1.333–5.899)	0.007*
OS		4.951 (2.457–9.979)	<0.001*	2.488 (1.006–6.152)	0.049*	7.233 (3.112–16.812)	<0.001*	5.787 (2.548–13.143)	<0.001*

^a^*p*-value by χ^2^ test. ^b^*p*-value by Kruskal–Wallis test with Bonferroni correction for the post-hoc test. *p-value < 0.05. OR, odds ratio; CI, confidence interval; SIS, segment involvement score; SSS, segment stenosis score; OS, obstructive stenosis; CT-LeSc, CCTA-adapted Leaman score.

## Discussion

In the UKPDS risk engine, age, duration of diabetes, male sex, ethnicity, current smoking, glycated hemoglobin, systolic blood pressure, and ratio of total cholesterol to HDL cholesterol are associated with the development of sudden death or myocardial infarction (29) as a basis for risk stratification.

In this study, we found that all four CCTA stenosis scores (SIS, SSS, SC, and SPP) and CT-LeSc were significantly higher in T2DM patients than those in non-DM non-CHD patients, which seems useful for CHD screening. However, no difference was observed between the low-risk T2DM patients and non-DM non-CHD patients when we divided the T2DM patients into three subgroups according to the CHD fatal risk, which was predicted by UKPDS risk engine. There was a significant difference between the moderate-high risk T2DM patients and non-DM non-CHD patients as before. The moderate-high risk T2DM patients had a higher risk of coronary artery stenosis and higher plaque burden than the patients without DM and CHD, which was shown in the higher OR of SIS > 3, SSS > 5, CT-LeSc > 8.7, and OS.

The SIS reflect account the number of segments with plaque, the SSS take into the degree of stenosis, the CT-LeSc quantify the overall plaque burden and takes into the localization and type of the plaques. Combining these scores can comprehensively evaluate the coronary atherosclerosis of the T2DM patients.

Compared with people without DM, the prevalence of CHD in T2DM patients is higher (30). According to the results of CCTA, the prevalence and severity of CHD in T2DM patients is higher than in those without DM (31). However, there are few data on the clinical significance of CCTA in asymptomatic T2DM patients.

In previous studies, it was observed that approximately 64–91.4% of asymptomatic T2DM patients had atherosclerosis, and 26–33.3% of patients had severe CHD (32–35). Consistent with previous studies, atherosclerotic plaques were found in 128 (90%) T2DM patients in this study and 69 (49%) patients had ≥50% luminal diameter stenosis. This suggests that CHD in asymptomatic T2DM patients is a problem that cannot be ignored. Twenty (14%) patients with T2DM developed severe proximal stenosis of multiple coronary arteries, which indicates a poor prognosis (26). In the high-risk group, the proportion was as high as 21%, indicating that the increased severity of CHD is associated with an increased fatal risk in T2DM patients (31, 36).

Currently, it is thought that T2DM patients are not always at the highest CHD risk state, nor do all T2DM patients have the same high CHD risk (37). According to an asymptomatic diabetic study that evaluated the detection of ischemia, the heart risk of patients with moderate and severe ischemia is six times higher than that of patients with normal or small perfusion defects (38). These findings suggest that we should try to identify high-risk patients, especially asymptomatic T2DM patients.

Significant differences were observed in all four CCTA stenosis scores and CT-LeSc between the control group and the moderate-high risk group. No difference was observed in any of the four CCTA stenosis scores and CT-LeSc between the control group and low-risk group. Approximately 28% of T2DM patients received little benefit from CCTA examination but had to bear unnecessary risks (i.e., X-ray radiation, iodine contrast agent allergy, and kidney injury). Using CCTA in the CHD screening of low-risk asymptomatic T2DM patients should not be recommended, which also supports the conclusion of previous studies (15, 16).

Compared with non-DM non-CHD patients, moderate-high risk T2DM patients have a higher risk of coronary artery stenosis, higher CCTA stenosis scores and higher overall plaque burden, which means more severe stenosis and worse prognosis. This finding demonstrates the screening role of CCTA in moderate-high risk asymptomatic T2DM patients using risk stratification prediction, especially in high-risk patients.

### Study limitations

This study is a single center retrospective study, subjects only represent a relatively single group, and there may be potential bias in the selection and test ability. The incidence and prognosis of CHD may be influenced by geographical factors, climate and the eating habits of subjects. The choice of patients without DM and CHD as the control group may not fully reflect the status of the whole healthy population.

This study did not systematically correlate the CCTA results with the ICA reference standards for intracoronary plaque assessment. Although the consistency effect of plaque post-processing software has been verified in other studies (39, 40), the scores of plaque may still be different in different automatic post-processing software programs. Therefore, the same post-processing software should be used in a series of studies. Likewise, the measurement of plaque stenosis may also be influenced by technology, e.g., by hardening-induced artifacts around severe calcified plaques and the attenuation level of vascular contrast enhancement (41).

United Kingdom prospective diabetes study Risk Engine 2.0 is a validated, easy to use tool to predict events in diabetic population, but it overlooks other important and well recognized cardiovascular disease risk factor such as chronic renal disease and peripheral artery disease. It also does not differ diabetes with target organ damage from well controlled diabetes. Additionally, there is a potential difference between the risk stratification predicted by the UKPDS risk engine and the actual risk in asymptomatic T2DM patients. Therefore, these results might be applicable to very specific populations, and prospective studies on larger populations will be necessary to validate our findings. We will conduct a long-term follow-up observation to verify the findings.

## Conclusion

The impact of DM on cardiovascular disease is well known. The incidence rate of CHD is higher in DM patients, and the prognosis is even worse. In asymptomatic T2DM patients, the diagnosis of CHD is usually missed or delayed, which in turn enhances the risk for cardiovascular events.

Through the UKPDS risk engine software, the moderate-high risk groups of asymptomatic T2DM patients may be screened out according to the risk stratification prediction of CHD. Using CCTA may effectively screen for coronary stenosis in corresponding patients.

It was observed in this study that coronary stenosis in moderate-high risk asymptomatic T2DM patients is more extensive and more serious than in non-DM non-CHD populations, and will result in more cardiovascular events. In these populations, early CCTA examination is necessary, which means early diagnosis of CHD and early treatment to ultimately reduce the incidence of cardiovascular events and improve prognosis.

## Data availability statement

The raw data supporting the conclusions of this article will be made available by the authors, without undue reservation.

## Ethics statement

The studies involving human participants were reviewed and approved by Review Board of the Affiliated Hospital of Jining Medical University. Written informed consent for participation was not required for this study in accordance with the national legislation and the institutional requirements.

## Author contributions

QL, JQ, and HZ designed the study. QL analyzed the data and wrote the first draft of the manuscript. JQ participated in the study design, analyzed the data, and edited and reviewed the manuscript. HZ supervised the overall study and contributed to the study design and editing and review of the manuscript. SS was responsible for collecting, sorting, and statistical data. XW and ZS were responsible for data collection, statistical analysis, and text modification of the revision. All authors have read and approved the final manuscript.

## Footnotes

1
http://www.dtu.ox.ac.uk/riskengine/

## Abbreviations

CCTA: coronary computed tomography angiography
CHD: coronary heart disease
CI: confidence interval
CT-LeSc: CCTA-adapted Leaman score
HDL: high-density lipoprotein
ICA: invasive coronary angiography
OR: odds ratio
OS: obstructive stenosis
SC: stenosis coefficient
SIS: segment involvement score
SPP: severe proximal plaque
SSS: segment stenosis score
T2DM: type 2 diabetes mellitus.
